# Recent advances in near-infrared dye conjugates for near-infrared photoimmunotherapy (NIR-PIT): enhancing therapeutic efficacy and immune mechanisms

**DOI:** 10.1039/d6cb00132g

**Published:** 2026-06-17

**Authors:** Yoshikazu Fuse, Eita Sasaki, Orie Takayama, Sota Yamada, Kenjiro Hanaoka

**Affiliations:** a Graduate School of Pharmaceutical Sciences, Keio University 1-5-30, Shibakoen, Minato-ku Tokyo 105-8512 Japan khanaoka@keio.jp; b Human Biology-Microbiome-Quantum Research Center (WPI-Bio2Q), Keio University Tokyo 108-8345 Japan

## Abstract

Near-infrared photoimmunotherapy (NIR-PIT) is an innovative cancer treatment modality that was approved in Japan in 2020 for the treatment of unresectable locally advanced or locally recurrent head and neck cancer. This therapy uses an antibody-dye conjugate (Ab-IR700), which consists of a monoclonal antibody targeting a specific cell-surface antigen and a phthalocyanine-based near-infrared dye, IR700, that functions as a photosensitizer. After selective accumulation in tumor tissue, Ab-IR700 is irradiated with 690 nm NIR light, which initiates a photochemical reaction that selectively damages the cell membrane of target cells, thereby inducing immunogenic cell death. Its high tumor selectivity and therapeutic efficacy establish NIR-PIT as a promising next-generation cancer therapy. However, its further application to deep-seated solid tumors remains challenging, and will require IR700 analogs and novel dye scaffolds that can be activated by longer-wavelength light to achieve greater tissue penetration and that offer greater photochemical activation efficiency. This review covers the activation mechanism of IR700, the mechanisms of cytotoxicity of NIR-PIT, emerging applications of NIR-PIT in oncology and infectious diseases, the range of dye delivery vehicles, and the development of new dyes for NIR-PIT.

## Abstract 2

NIR-PIT is a cancer therapy using antibody-IR700 conjugate. This review covers IR700's light-induced activation, cytotoxic mechanisms, analogs, and delivery vehicles. It further explores clinical applications and its potential in disease treatment.

## Graphical abstract

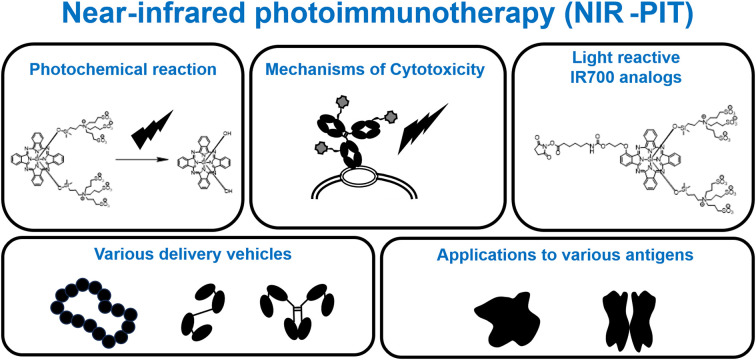

## Introduction

Photodynamic therapy (PDT) is a cancer treatment that utilizes dye molecules known as photosensitizers, which are activated by light irradiation.^1^ Near-infrared (NIR) light is particularly well suited for this purpose due to its high tissue-penetrating ability. In PDT, cell damage is induced by reactive oxygen species (ROS) generated through two distinct pathways following light irradiation of the photosensitizer. In Type I reactions, the excited photosensitizer generates radical species, including superoxide, hydrogen peroxide, and hydroxyl radicals, through electron or hydrogen transfer processes. In Type II reactions, energy transfer from the excited photosensitizer to molecular oxygen generates singlet oxygen, which induces oxidative cellular damage.^2,3^ While PDT has gained significant attention, several issues remain, such as weak absorption of photosensitizers in the red-to-NIR region, low cancer specificity and oxygen dependence.^4^ For example, clinically approved talaporfin sodium (Laserphyrin®) exhibits Q-band absorption at approximately 664 nm in the visible red region. Although this wavelength is suitable for PDT, deeper tissue penetration would require absorption at longer wavelengths within the biological optical window, where endogenous chromophores such as haemoglobin exhibit lower absorption. Thus, there is a need to develop photosensitizers with strong absorption in the NIR region to improve the tissue penetration and efficacy of PDT. Photosensitizer delivery systems based on nanoparticles or macromolecules can utilize the enhanced permeability and retention (EPR) effect for tumor accumulation.^5^ However, this approach is limited by the heterogeneity of the tumor microenvironment, even within a single individual, and off-target effects on healthy tissues also remain a concern. Although antibody–photosensitizer conjugates have been explored to improve targeting, antibody conjugation can reduce cellular internalization. Since many photosensitizers exert stronger cytotoxicity after cellular internalization, this remains a key challenge in PDT.^6,7^

To address these issues, near-infrared photoimmunotherapy (NIR-PIT) using an antibody-IR700 conjugate consisting of a monoclonal antibody labeled with the NIR fluorescent dye IRDye700DX® (IR700) which was developed and patented by LI-COR company has recently been developed as a novel light-based cancer treatment.^8–10^IR700 is a phthalocyanine (Pc)-based dye with strong Q-band absorption, enabling efficient excitation by 690 nm NIR light, which exhibits relatively high tissue penetration.^11^ Further, since IR700 contains highly hydrophilic moieties, dye labeling has little effect on the stability of the antibody.^11^

In NIR-PIT, the excitation of IR700 by NIR light irradiation induces cell death through physical membrane damage that is basically independent of singlet oxygen, thereby reducing damage to surrounding normal cells.^11,12^ This cytotoxicity is caused by an irreversible change of IR700 from a hydrophilic to a hydrophobic state in response to irradiation. In addition, the cell damage caused by NIR-PIT induces an immune response,^12^ which produces an abscopal effect, where cell death occurs *via* an immune response even in distant, non-irradiated cancer cells. Although immune responses have also been reported in PDT, the mechanism underlying these responses differs from that in the case of NIR-PIT. NIR-PIT also affords increased drug permeability into the tumor.^13^ Due to its high cancer specificity, NIR-PIT was approved in 2020 for the treatment of unresectable locally advanced or recurrent head and neck cancer in Japan, and clinical trials for other cancers are currently underway. At present, IR700 is the only NIR fluorescent dye available for NIR-PIT in clinical practice. Although the 690 nm light used in NIR-PIT has relatively high tissue penetration, longer-wavelength light generally provides greater tissue penetration.^14,15^ Therefore, developing dyes with red-shifted excitation wavelengths is desirable for treating deeper or larger tumors. Various approaches are being explored to expand the range of available NIR dye molecules. Here, we review (1) the activation mechanism of IR700, (2) the mechanism of the cytotoxicity of NIR-PIT with IR700, (3) applications of NIR-PIT using IR700, (4) applications employing various dye delivery vehicles, and (5) recent progress in the development of new dyes for NIR-PIT.

## Activation mechanism of IR700

IR700 is a silicon phthalocyanine incorporating hydrophilic axial ligands attached to a hydrophobic phthalocyanine core. Cytotoxicity in NIR-PIT is triggered by light-induced cleavage of the hydrophilic axial ligands of IR700.^11^

**Fig. 1:**
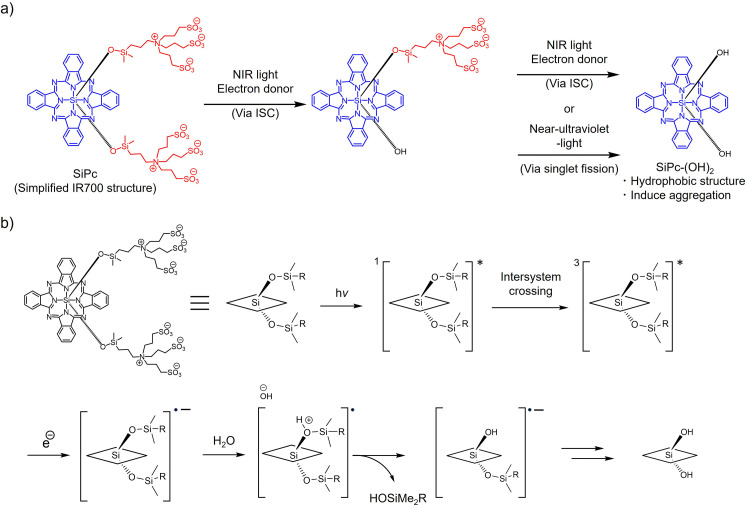
(a) Overview of the photochemical reaction of IR700. (b) Mechanism of cleavage of axial ligands in the silicon phthalocyanine compound.

Fig. 1a shows a schematic representation of the light-induced activation mechanism of IR700 without the linker moiety (SiPc).^16–19^ Upon light irradiation, an axial ligand is cleaved *via* an intersystem crossing (ISC) pathway in the presence of an electron donor. The second axial ligand is then cleaved *via* either the ISC pathway or a singlet fission pathway to generate SiPc-(OH)_2_. This highly hydrophobic structure forms aggregates, inducing cytotoxicity.

From a photophysical perspective, this axial ligand cleavage is initiated by transition of photoexcited IR700 from its singlet excited state (S_1_) to a triplet excited state (T_1_) *via* ISC with a relatively low probability.^16^ While the lifetime of the singlet excited state is extremely short (approximately ∼10^−9^ s), the triplet excited state has a much longer lifetime (10^−6^ to 10^−3^ s), providing sufficient time for chemical reactions to proceed. The intersystem crossing quantum yield (*Φ*_ISC_) of an IR700 model compound was calculated to be approximately 0.019.^17^ The formation of the triplet excited state enables electron transfer from a donor to the dye, generating an IR700 radical anion.^18^ The reaction proceeds in the absence of oxygen, which acts as a triplet quencher, supporting the involvement of the triplet state^18^ (Fig. 1b).

Moreover, the more complex photophysical phenomenon of singlet fission has been suggested to occur after the cleavage of the first axial ligand. Tsuneda *et al.* found that when the first ligand is cleaved and replaced by an OH group, the distance between phthalocyanine molecules decreases, leading to aggregation *via* π–π stacking.^19^ In this stacked state, high-energy photoexcitation may promote singlet fission, generating two triplet states from a single excited state.^19^ This could provide the triplet state for cleavage of the second axial ligand.

The chemical dissociation process has been elucidated in detail by Kobayashi *et al.* through density functional theory (DFT) calculations and mass spectrometry using ^18^O-labeled water. After the silicon phthalocyanine enters the excited triplet state and accepts an electron from a nearby electron donor to form a radical anion, a proton from the surrounding water or the environment attaches to the oxygen atom of the siloxy group. This protonation represents the highest energy barrier in the reaction pathway. However, once protonated, the silicon (Si)–oxygen (O) bond elongates and dissociates, and the detachment of the axial ligand is completed upon the addition of a water molecule (Fig. 1b).^16^ This reaction proceeds more readily at low pH, suggesting that the tumor microenvironment would influence the reaction efficiency. In this way, IR700 converts light absorption into a sequence of spin-state transitions and chemical bond rearrangements, ultimately leading to the membrane-disruptive cytotoxicity observed in NIR-PIT.

## Mechanisms of NIR-PIT cytotoxicity

### Physical membrane disruption in NIR-PIT

**Fig. 2:**
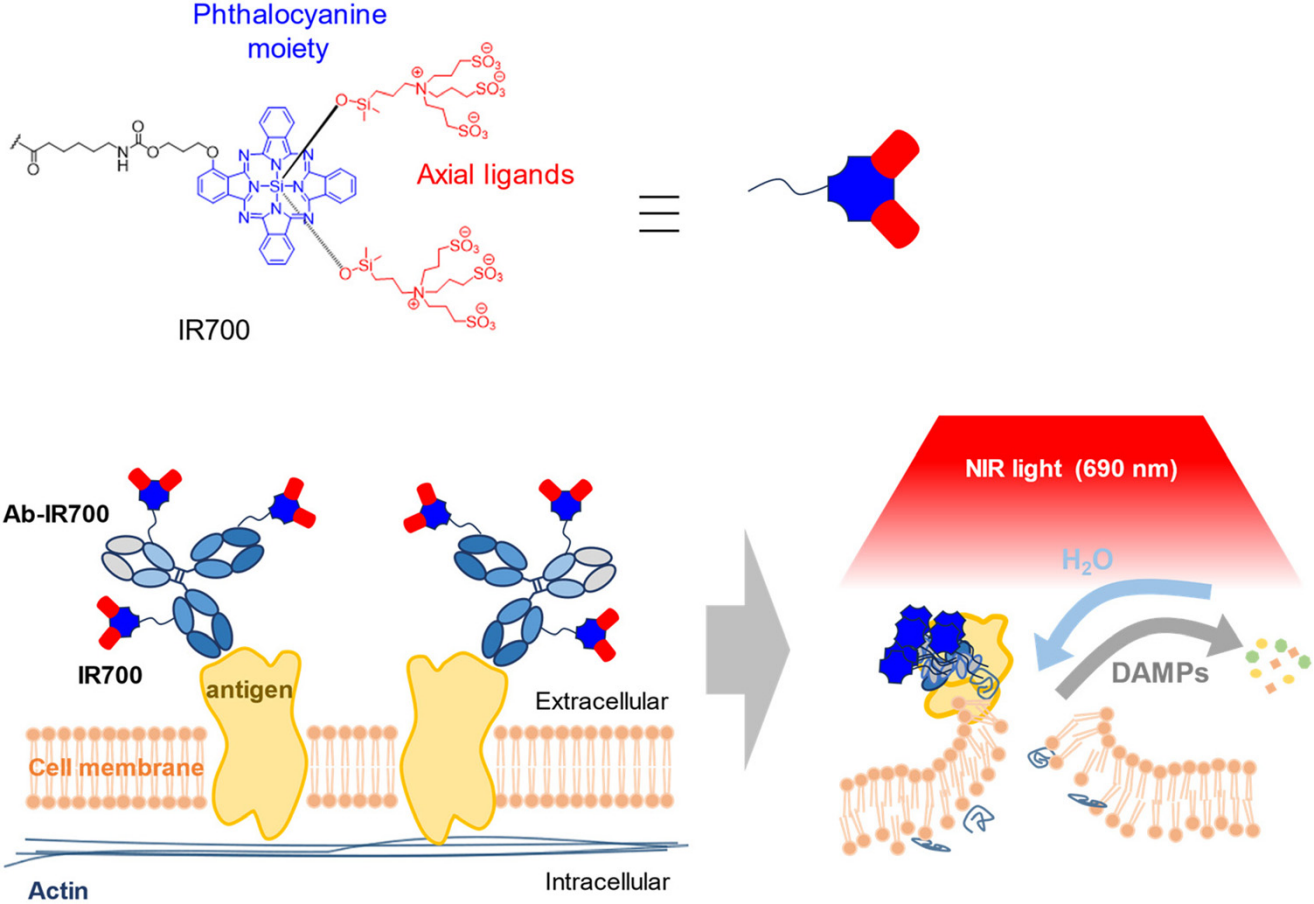
Proposed mechanism of NIR-PIT. NIR light irradiation activates IR700, leading to the aggregation of antigens, antibodies, and IR700 on the cell membrane, which results in cell membrane damage.

The proposed mechanism of NIR-PIT utilizes the unique photochemical properties of IR700 (Fig. 2).^10,11^ Initially, the administered antibody-IR700 conjugate (Ab-IR700) accumulates on the target cells through binding to specific cell-surface antigens. Upon irradiation with 690 nm NIR light, IR700 is activated, leading to the cleavage of its hydrophilic axial silanol ligands. This photochemical reaction exposes the highly hydrophobic phthalocyanine core, inducing the formation of water-insoluble aggregates, including silicon-phthalocyanine Z-stack multimers, Ab-IR700 and Ab-IR700-antigen complexes. These physical changes generate mechanical stress at the cell membrane, directly causing cell membrane damage that rapidly leads to cell death.^11,20^

The actin cytoskeleton also plays a critical role in the mechanism of NIR-PIT-induced cell death.^20^ Immunoprecipitation experiments demonstrated that receptors such as human epidermal growth factor receptor 2 (HER2) and epidermal growth factor receptor (EGFR) form complexes with β-actin, which anchors these receptors to the cortical actin network beneath the cell membrane.^21,22^ Upon NIR light irradiation, the antibody–antigen complex forms massive aggregates. During this process, the associated actin filaments are physically entangled and pulled into the aggregates, destroying the normal network structure. Since cortical actin is the central structure for maintaining membrane tension and shape, its destruction renders the membrane unable to withstand external pressure. This collapse of the cortical structure was observed by means of scanning electron-assisted dielectric microscopy (SE-ADM).^23,24^ Actin polymerization inhibitors significantly reduced the rate of cell death.^20^

Along with membrane damage, cell swelling and rupture were observed following photoirradiation. Three-dimensional dynamic low-coherence quantitative phase microscopy (3D LC-QPM)^25,26^ revealed that cells treated with anti HER2 antibody (trastuzumab)-IR700 conjugate began to swell just five seconds after exposure to NIR light, and reached their maximum volume within one minute.^11^ Conversely, when cells were placed in a high-osmolarity buffer containing 50 mM dextran, the cell swelling was suppressed, indicating that the cell rupture is caused by influx of water driven by the osmotic pressure gradient following membrane damage.^27^ During this process in isotonic solution, the cells swelled to approximately three times their original volume before bursting. Further, the enhanced uptake of charged molecules and macromolecules across the cell membrane also supports the idea that the cell membrane is damaged. To visualize the release of the intracellular contents after photoirradiation, dual-view inverted selective plane illumination microscopy (diSPIM) was employed.^28,29^ The rapid cell swelling induces large tears in the cell membrane, leading to the expulsion of cytoplasmic molecules into the extracellular space. Observations in cells expressing cytoplasmic green fluorescent protein (GFP) showed that the protein was retained during the initial swelling phase but was rapidly released upon membrane rupture, accompanied by a sudden decrease in cell volume. Following this rupture, charged molecules such as hydrolyzed calcein-AM and ethidium homodimer-1 can move freely across the impaired membrane.^12^

The role of singlet oxygen in NIR-PIT was also investigated. Although the addition of sodium azide, a singlet oxygen quencher, failed to inhibit cell rupture, suggesting that cell death is largely independent of singlet oxygen, the complete absence of oxygen significantly suppressed membrane damage.^30^ Given that this damage occurs even at 4 °C, the process is likely non-enzymatic, possibly involving the oxidation of membrane lipids.^10,11^ These findings indicate that while oxygen may not be the primary driver of cell death, it may serve as a trigger or facilitator for the initial stages of the cytotoxic reaction.

### Determinants of NIR-PIT efficacy and early response assessment

While evidence for the core cytotoxic mechanism of NIR-PIT is robust, therapeutic outcomes can vary depending on multiple biological factors, such as intracellular trafficking, the cell state and the tumor microenvironment. This section summarizes key determinants of cytotoxic efficacy and practical readouts used to assess early responses *in vitro* and *in vivo*.

Although the generation of ROS is not essential for Ab-IR700-triggered membrane disruption at the cell surface, it has been reported that internalized Ab-IR700 conjugates induce necrotic cell death by disrupting the lysosomal compartment *via* ROS generation.^31^ Therefore, the selection of target molecules and the precise control of their cellular localization are important for optimizing therapeutic efficacy.

The correlation between NIR-PIT cytotoxicity and the cell cycle has been analyzed using a fluorescent ubiquitination-based cell cycle indicator (Fucci).^32,33^ Research using human squamous cell carcinoma (CAL33, HSC3) and cervical cancer (HeLa) cell lines has established that NIR-PIT-induced cytotoxicity is cell-cycle dependent. Studies utilizing cetuximab (anti-EGFR antibody)-IR700 demonstrated that cells in the G1 phase exhibit higher resistance and slower progression of cell death compared to those in the S, G2, and M phases. This difference in therapeutic efficacy correlates with the fluctuations of EGFR expression levels throughout the cell cycle. Consequently, these findings indicate that the effectiveness of NIR-PIT is determined by the target antigen density at the time of irradiation.^34^

*In vivo* PET/CT imaging with ^18^F-FDG, an indicator of glucose metabolism, suggests that NIR-PIT causes an acute attenuation of glucose metabolism within cancer cells, resulting in cell death.^35^ In addition, an analysis using ^18^F-fluoromisonidazole-PET confirmed that light-induced activation of Ab-IR700 alleviated hypoxia within the tumor core.^35^ Thus, by integrating various imaging modalities, it is possible to evaluate the therapeutic efficacy and biological responses elicited by NIR-PIT from various aspects.

### Immune activation induced by NIR-PIT

**Fig. 3:**
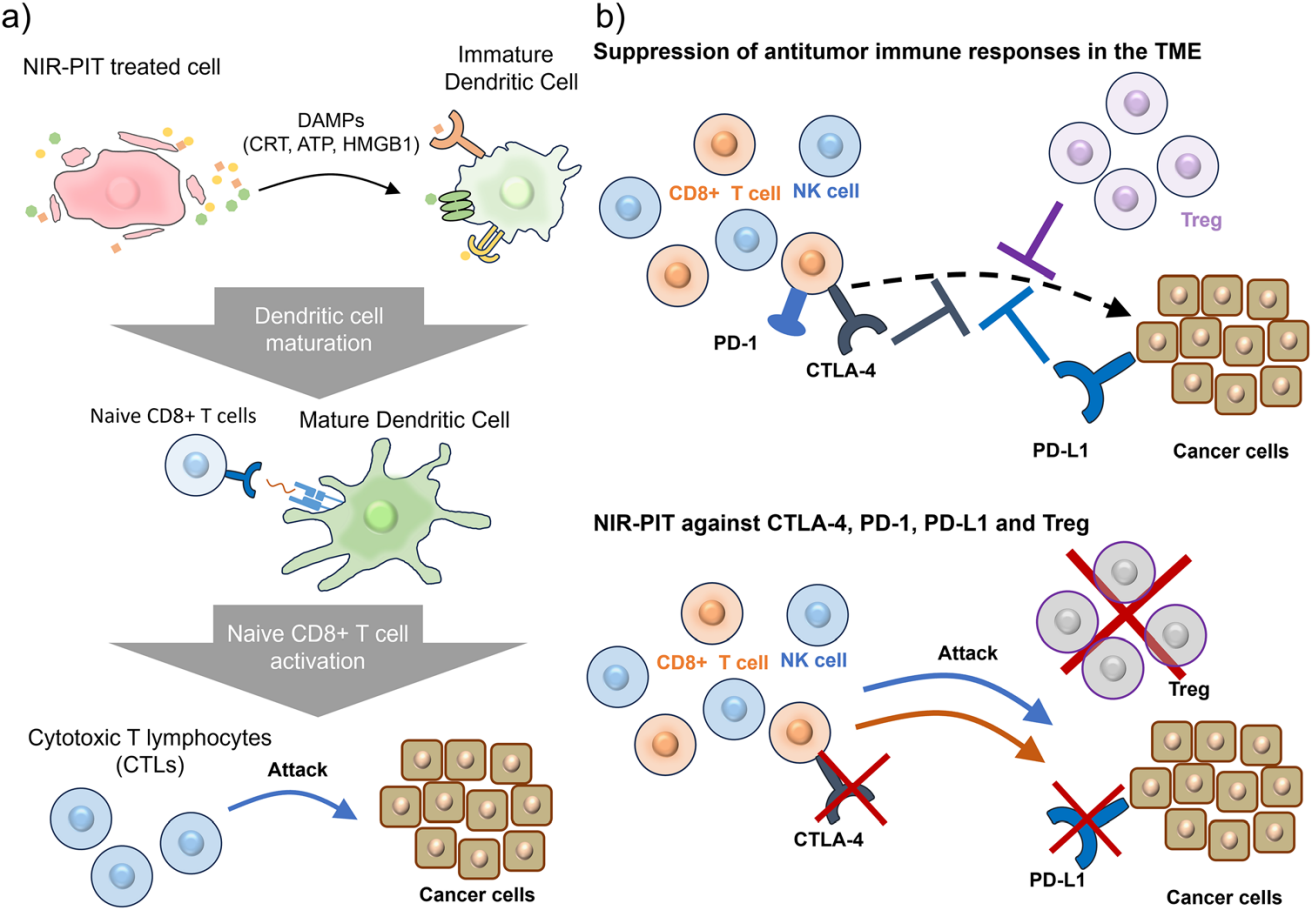
(a) Activation of anti-tumor immunity following NIR-PIT. (b) Immune activation *via* NIR-PIT targeting Treg or immune checkpoint.

NIR-PIT also induces immunogenic cell death, actively promoting the maturation of immature dendritic cells (DCs) within the tumor microenvironment.^12^ Cells treated with NIR-PIT undergo rapid cell rupture, followed by activation of stress markers, including heat shock proteins 70 (Hsp70) and 90 (Hsp90),^36,37^ and release of danger signals such as calreticulin, ATP and high-mobility group box 1 (HMGB1). These damage-associated molecular patterns (DAMPs) serve as biochemical signals that activate adjacent immature DCs, facilitating their differentiation into a mature state with increased surface expression of CD80, CD86 and HLA-DR. These mature DCs then process tumor-associated antigens released during cell lysis and present them to naive T cells, a process known as priming. This process generates activated cytotoxic T lymphocytes (CTLs) capable of recognizing and destroying other cancer cells throughout the body (Fig. 3a).^12^ Thus, although the cytotoxic effect of NIR-PIT may degrade malignant cells within a tumor, the subsequent host immune response can effectively eliminate the remaining cells. Although PDT is also known to induce immunogenic cell death, the underlying mechanism differs in principle: PDT induces immunogenicity through ROS-mediated oxidative damage, whereas NIR-PIT induces it through the release of DAMPs following rapid membrane damage.^3,4^

Moreover, NIR-PIT enhances the host immune response by selectively depleting immunosuppressive cells within the tumor microenvironment, utilizing Ab-IR700 conjugates that target specific markers such as CD25 on regulatory T cells (Tregs) or Gr-1 on myeloid-derived suppressor cells (MDSCs).^38–40^ The efficacy of NIR-PIT is also enhanced when conducted in combination with immune checkpoint inhibitors such as PD-1/PD-L and CTLA-4, or by employing NIR-PIT with IR700-labeled anti-PD-L1 or anti-CTLA-4 antibody to locally activate CD8+ T cells (Fig. 3b).^41–44^

### Super-enhanced permeability and retention (SUPR) effect

**Fig. 4:**
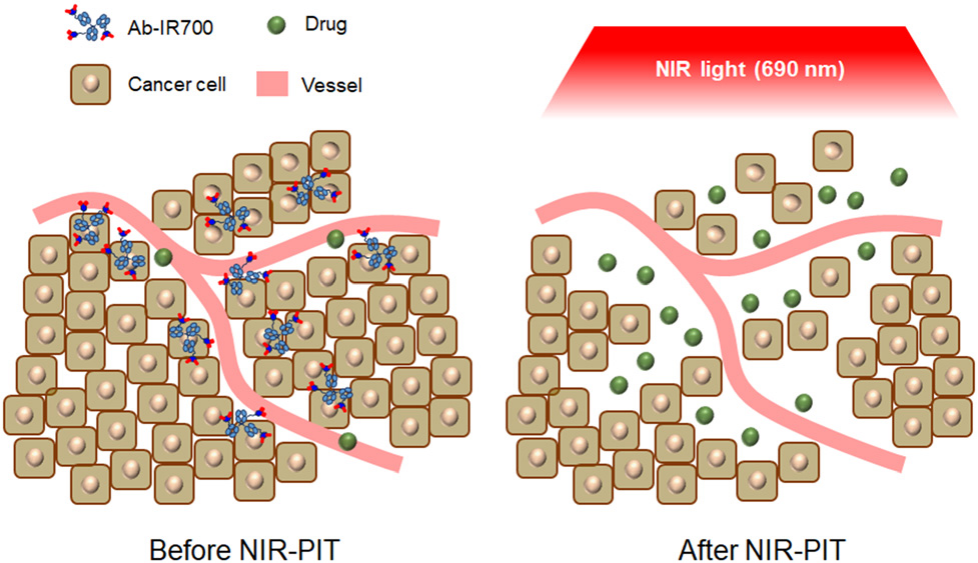
SUPR Effect after NIR-PIT.

A key pharmacological property of NIR-PIT is its ability to acutely enhance drug delivery to tumor tissues. In general, the accumulation of macromolecules in solid tumors relies on the EPR effect, which arises from the abnormal and leaky nature of tumor vasculature. However, immediately following NIR-PIT, a rapid increase in vascular permeability is observed, exceeding the conventional EPR effect. This phenomenon is called the super-enhanced permeability and retention (SUPR) effect (Fig. 4).^13^ It is triggered by the rapid physical rupture of perivascular cancer cells upon light irradiation. This induces structural gaps between vessel walls and the tumor tissue, leading to a decrease in interstitial pressure. The resulting pressure gradient, combined with vascular expansion and decreased blood flow, facilitates the extravasation and prolonged retention of macromolecules within the tumor.^13^

There is experimental evidence of the potency of the SUPR effect; for instance, NIR-PIT has been shown to increase the accumulation of 50 nm quantum dots by up to 25.7-fold and that of liposomal daunorubicin (mean diameter, 50 nm) by over 10-fold.^13,45^ Further, by eliminating perivascular cells, NIR-PIT may overcome the binding-site barrier, thereby enabling a more uniform distribution of subsequently administered antibody therapies such as antibody–drug conjugates throughout the tumor core.^46^ This dynamic enhancement of organizational transparency could be non-invasively monitored by various imaging methods, including fluorescence imaging with indocyanine green (ICG), contrast-enhanced MRI and contrast-enhanced ultrasound.^47^

## Applications of NIR-PIT using IR700

### Applications in oncology and other diseases

In Japan, NIR-PIT using a cetuximab (anti-EGFR antibody)-IR700 conjugate (Akalux®) is approved for clinical use as a treatment for unresectable locally advanced or recurrent head and neck cancer.^48^ Akalux® is administered by intravenous infusion at a dose of 640 mg m^−2^ over at least 2 hours, followed by laser irradiation of the lesion site 20–28 hours after completion of the infusion. NIR light at 690 nm is delivered using the BioBlade Laser System (Rakuten Medical, Tokyo, Japan). For superficial lesions, external irradiation is performed at a power density of 150 mW cm^−2^ with a total light dose of 50 J cm^−2^. In contrast, for interstitial irradiation of deep lesions, a diffuser is inserted into the tumor, and irradiation is performed at a power density of 400 mW cm^−2^ with a total light dose of 100 J cm^−2^.^49^ At the basic research level, numerous studies have reported applications targeting a variety of antigens, primarily EGFR and HER2, across diverse malignant tumors (Table 1). In addition to these tumor cell-directed targets, some approaches have focused on targeting cancer stem cell markers (*e.g.*, CD44 and CD133), and tumor microenvironment components, or on developing combination strategies with surgery or immune checkpoint blockers.^97^

**Table 1:** Applications of NIR-PIT to treat various cancers

Cancer	Antigen	Ref.
Head and neck cancer	EGFR	50–53
Glioblastoma	EGFR	54
Glioblastoma	CD133	55
Neuroblastoma	Disialoganglioside (GD2)	56
Lung cancer	EGFR	57
Lung cancer	Delta-like protein 3	58
Lung cancer	HER2	59 and 60
Malignant pleural mesothelioma	Podoplanin	61
Breast cancer	EGFR	62
Breast cancer	Intercellular adhesion molecule-1	63 and 64
Breast cancer	CD44	65
Gastrointestinal cancer	Cadherin-17	66
Gastric cancer	HER2	67
Gastric cancer	Carcinoembryonic antigen	68
Esophageal carcinoma	EGFR	69, 70 and 73
Esophageal carcinoma	HER2	69, 71 and 73
Esophageal carcinoma	Cancer-associated fibroblasts (CAFs)	70–72
Esophageal carcinoma	Epithelial cell adhesion molecule	73
Colorectal cancer	Carcinoembryonic antigen (CEA)	74
Colorectal cancer	Glycoprotein A33 antigen	75
Colorectal cancer	EGFR	76
Hepatocellular carcinoma	Glypican-3	77
Pancreatic carcinoma	Tumor-associated calcium signal transducer 2	78
Pancreatic carcinoma	CEA	79–81
Pancreatic carcinoma	CD44	82
Pancreatic carcinoma	CAFs	83
Renal cell carcinoma	Carbonic anhydrase-9	84
Bladder cancer	EGFR	85
Bladder cancer	HER2	85
Bladder cancer	CD47	86 and 87
Ovarian cancer	HER2	88
Ovarian cancer	EGFR	89
Prostate cancer	Prostate-specific membrane antigen	90
B cell lymphoma	CD20	91 and 92
Osteosarcoma	GD2	93
Osteosarcoma	EGFR	94
Cancer stem cells	CD271	95
Senescent cancer	HER2	96
Senescent cancer	EGFR	96

### Applications in infectious diseases

Beyond oncology, NIR-PIT is also applied to treat bacterial, fungal and viral infections. In studies targeting *Staphylococcus aureus* (*S. aureus*) with a peptidoglycan-specific antibody-IR700 conjugate, the treatment achieved selective elimination without damaging host cells or non-targeted microbiota. Mechanistically, although the antibacterial activity was slightly reduced in the presence of ascorbic acid, the reduction was less than 1%, suggesting that singlet oxygen is not the primary mediator of the antibacterial effect. Furthermore, catalase treatment did not inhibit antibacterial activity, indicating that hydrogen peroxide is not critically involved. Electron microscopy revealed membrane disruption with minimal detectable cell debris, suggesting that the antibacterial effect is mediated primarily through a membrane damage mechanism similar to that of NIR-PIT rather than ROS-dependent cytotoxicity.^98^

The application of NIR-PIT has also extended to fungal pathogens such as *Candida albicans* (*C. albicans*). Targeting mannan epitopes with an IR700-conjugated antibody resulted in selective killing of *C. albicans*, and an IR700-conjugated IgY antibody targeting *C. albicans* also facilitated recovery from persistent candidiasis in mouse models of skin wounds.^98,99^

As for viral infections, NIR-PIT has been successfully applied to treat SARS-CoV-2, HTLV-1 and influenza A. By targeting viral envelope proteins such as the SARS-CoV-2 spike protein, HTLV-1 gp46 and influenza hemagglutinin, the selective elimination of infected cells and the direct inactivation of free viruses have been achieved.^98,100,101^ Thus, these results suggest potential efficacy of NIR-PIT against diverse pathogens (Table 2).

**Table 2:** Applications of NIR-PIT for infectious diseases

Pathogen	Antigen	Ref.
MRSA	SA-cell wall peptidoglycan epitope	98
*Candida albicans*	CA-cell wall mannan epitope	98 and 99
SARS-CoV-2	Spike protein	98
HTLV-1	gp46	100
Influenza and infected cells	Hemagglutinin	101

## Applications with various delivery vehicles

### Engineered antibody formats and protein scaffolds

**Fig. 5:**
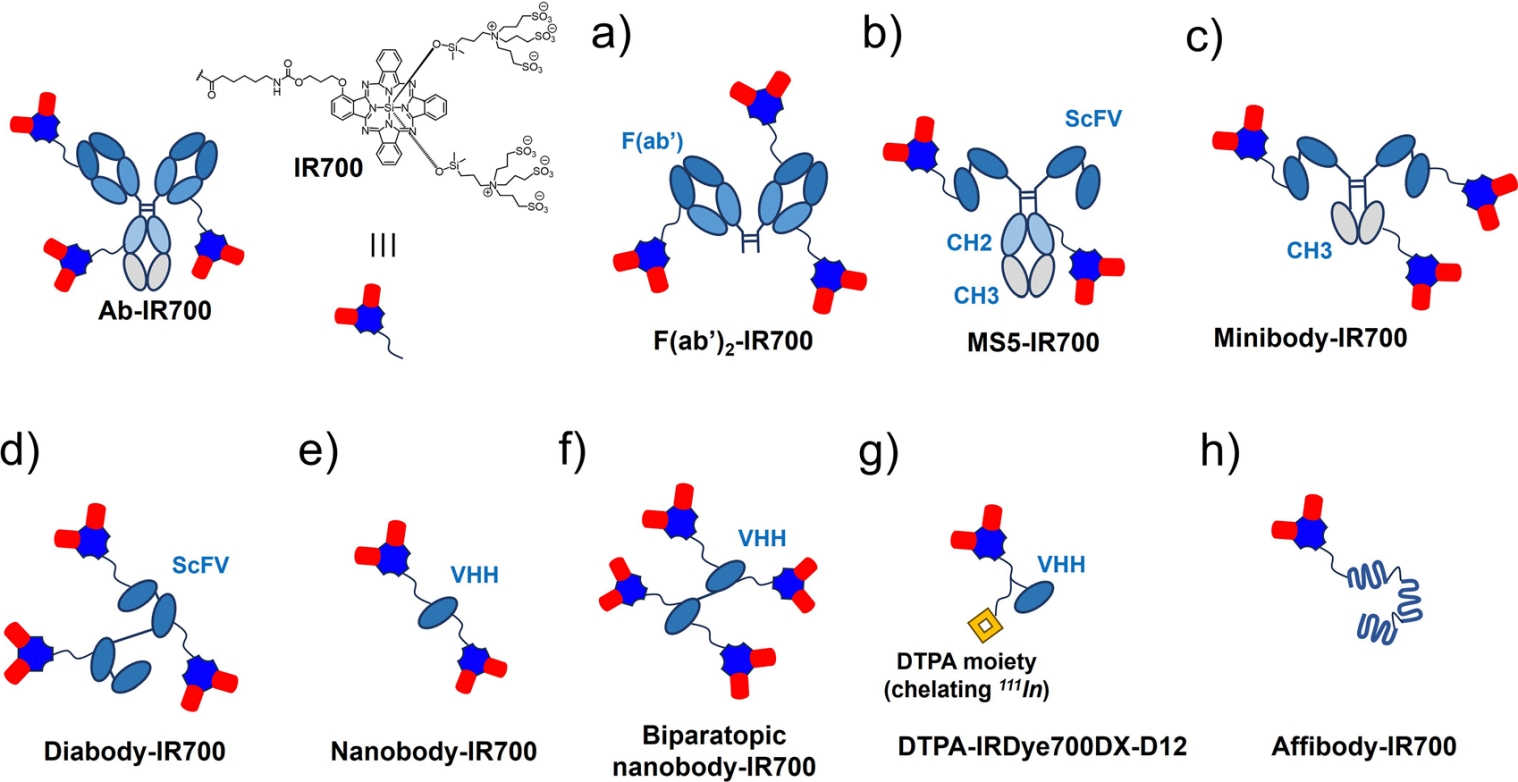
Engineered antibody format-IR700 conjugates. (a–f) F(ab′)_2_-fragments (a), single-chain variable fragment (scFv) fused to the CH2 and CH3 domain of IgG (b), scFV fused to the CH3 domain (c), two scFvs connected molecules (d), isolated variable domains (VHH) of heavy-chain-only antibody (e) and VHH dimer (f) were labeled with IR700. (g) VHH was labeled with the chelator diethylenetriaminepentaacetic acid (DTPA) and IR700. (h) Non-antibody-derived scaffold protein was labeled with IR700.

Recent research has explored diverse delivery vehicles for NIR-PIT, extending beyond conventional IgG antibodies. For example, F(ab′)_2_ fragments are generated by removing the Fc region from intact IgG antibodies. Therefore, they avoid off-target binding mediated by Fc receptors, and their shorter biological half-life may also reduce non-specific toxicity.^102^ NIR-PIT using IR700-labeled F(ab′)_2_ fragments targeting CD25 and CTLA-4 has been reported (Fig. 5a). The F(ab′)_2_ fragments were prepared by antibody digestion using immobilized ficin or lysyl endopeptidase. The F(ab′)_2_-based NIR-PIT agents were generated by conjugation with IR700 NHS ester through amino groups. Approximately three **IR700** molecules were conjugated per CD25-targeting F(ab′)_2_ molecule, whereas approximately four IR700 molecules were conjugated per CTLA-4-targeting F(ab′)_2_ molecule. In mouse models, NIR-PIT using F(ab′)_2_ is more effective than the IgG-based approach.^103–105^

Another approach is to use minibodies. A minibody is composed of a single-chain variable fragment (scFv) fused to the CH3 domain of IgG. Possessing a blood half-life intermediate between those of IgG and F(ab′)_2_ fragments, minibodies are particularly suitable for molecular imaging, such as immuno-PET, where rapid visualization after administration is required.^106,107^ MS5 is an engineered minibody analog composed of an scFv fused to CH3 domains and additional CH2 domains. IR700 NHS ester was conjugated to the MS5 at a labeling ratio of 1–2 IR700 molecules per MS-5 molecule, and the resulting conjugate induced light-mediated tumor cell death *in vitro* (Fig. 5b).^108^MS5–IR700 also penetrated deeply into the core of tumor spheroids and significantly inhibited their growth. Similarly, a prostate-specific membrane antigen (PSMA)-targeting minibody was conjugated with 2–4 IR700 molecules per minibody, and its cytotoxic effects towards PSMA-expressing cells were evaluated *in vitro* and *in vivo* (Fig. 5c).^109^ These results suggest that the smaller molecular size of minibodies relative to IgG facilitates access to the inner regions of tumor masses.

In contrast, diabodies are approximately 50 kDa molecules consisting of two scFvs connected by a short linker; unlike monovalent F(ab′)_2_ fragments, diabodies possess bivalent binding capacity.^106^ This allows them to exhibit high tissue permeability while maintaining strong antigen binding through the avidity effect; the multiple intermolecular interactions enhance the overall binding stability.^110^IR700 NHS ester-labeled diabodies targeting a PSMA mouse model have also been reported. Diabody molecules were conjugated with 2–4 IR700 molecules per molecule (Fig. 5d).^109^ The smaller diabody achieves faster tumor penetration than IgG or Mb, enabling irradiation just 6 hours post-injection. Crucially, Diabody enables the earliest initiation of treatment onset while showing equivalent therapeutic efficacy.^106^

Additionally, nanobodies are derived from the isolated variable domains (VHH) of heavy-chain-only antibodies. Characterized by a remarkably low molecular weight of approximately 15 kDa, their primary advantage lies in superior tumor tissue permeability, which is facilitated by their small size.^111^ Studies of EGFR-targeting VHHs have utilized the 7D12 nanobody, which recognizes EGFR domain III, and the biparatopic 7D12-9G8 nanobody, a VHH dimer capable of simultaneously binding two distinct epitopes at the boundary between EGFR domains II and III. These nanobodies were produced in *E. coli* and conjugated with IR700 for *in vitro* studies.^112^ In subsequent studies, approximately two IR700 molecules per 7D12 molecule and four IR700 molecules per 7D12-9G8 molecule were used to induce rapid antitumor effects *in vivo*, with the biparatopic nanobody exhibiting enhanced therapeutic efficacy (Fig. 5e and f).^113^

Nanobody-based NIR-PIT has also been applied to virus-associated targets. IR700-maleimide was conjugated at an average ratio of 0.7 dye molecules per nanobody to VUN100-Cys, which recognizes the virus-encoded G protein-coupled receptors (GPCRs) US28 and carries an engineered cysteine residue at its C-terminus. This construct selectively targeted US28-positive glioblastoma cells, even in 3D spheroid models.^114^

Furthermore, multifunctional nanobody conjugates combining imaging and therapeutic capabilities have been developed. **DTPA-NH-Tz-*****S*****-IRDye700DX**, which contains the chelator diethylenetriaminepentaacetic acid (DTPA) and IR700, was conjugated to the EGFR-targeting 7D12 nanobody at an average ratio of 0.56 molecules per nanobody (Fig. 5g). The ^111^In-chelating construct enabled simultaneous light-dependent cytotoxicity and SPECT imaging.^115^

Affibodies, which are non-antibody-derived scaffold proteins, are characterized by an extremely small molecular weight of approximately 6.5 kDa. Composed of a stable three-alpha-helix bundle, affibodies diffuse rapidly into the deep regions of tumors that are inaccessible to full-length antibodies, yet they are cleared from the bloodstream very quickly.^116^ Z_EGFR:03115_, an affibody that targets EGFR, was conjugated with IR700-maleimide at an average labeling ratio of 0.84 IR700 molecules per affibody. Similarly, Z_HER2:2395_-Cys, which targets HER2, was also conjugated with IR700-maleimide. These conjugates showed antitumor effects against EGFR- or HER2-expressing cells, respectively, both *in vitro* and *in vivo* (Fig. 5h).^117,118^

### Low-molecular-weight ligands and peptides

**Fig. 6:**
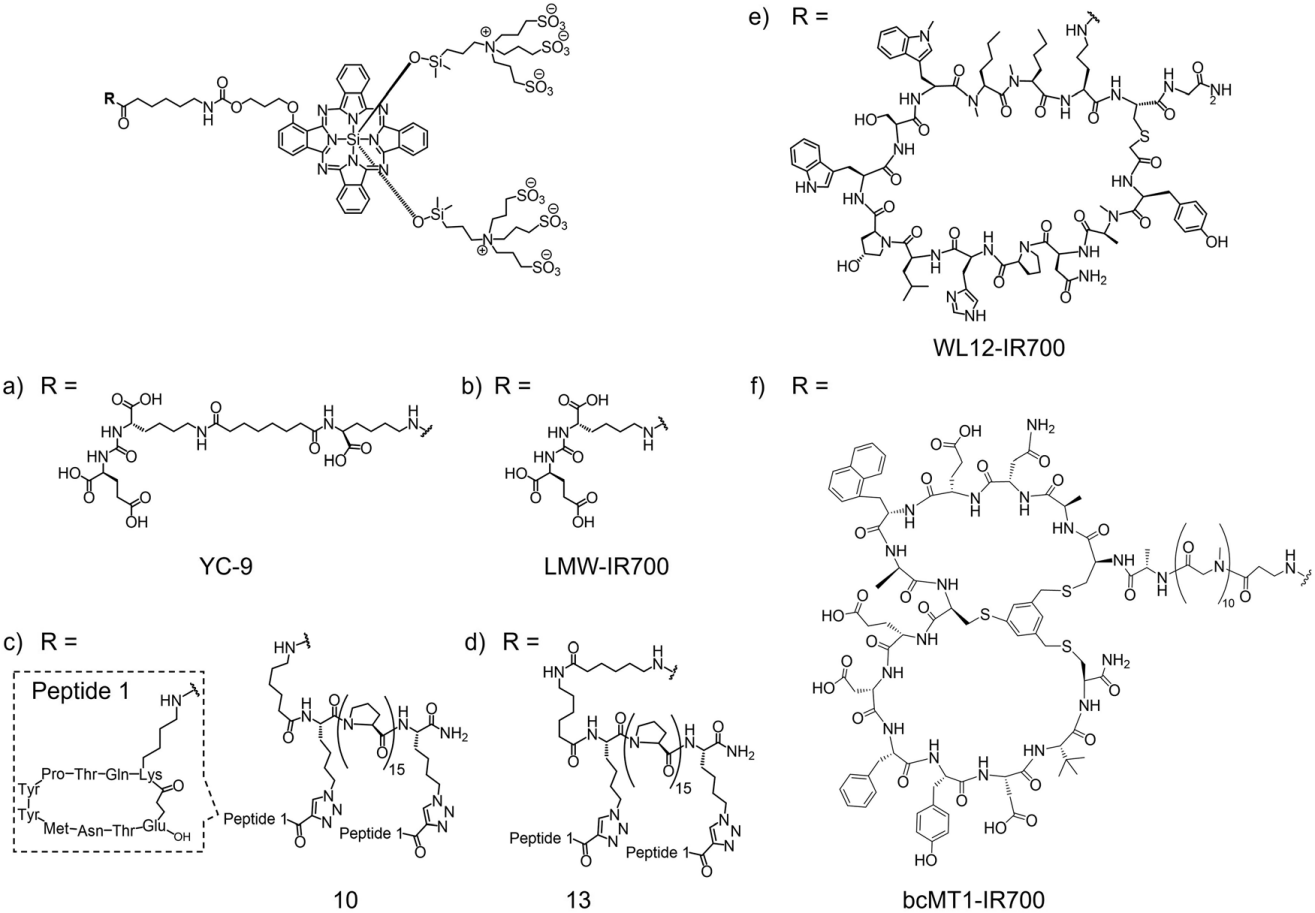
Peptide-IR700 conjugates. (a, b) PSMA-specific peptide ligand with (a) or without (b) the linker was conjugated with IR700. (c, d) EGFR binding peptide with the short (c) or long (d) linker was conjugated with IR700. (e, f) PD-L1-targeting (e) and membrane-type 1 matrix metalloproteinase targeting (f) (bi)cyclic peptides were conjugated with IR700.

To address prostate cancer, a low-molecular-weight ligand-IR700 conjugate named YC-9 was developed, comprising the PSMA-specific ligand Glu-Urea-Lys, a lysine-suberate linker and IR700 (Fig. 6a). YC-9 demonstrated PSMA-selective accumulation and induced light-dependent cell death in PSMA-expressing LNCaP cells upon NIR light irradiation. Further *in vivo* studies using mouse models showed that YC-9 administration followed by light irradiation inhibited tumor growth and prolonged survival.^119^ In a comparative study, LMW-IR700, in which IR700 is directly conjugated to the Glu-Urea-Lys ligand without the linker, was evaluated (Fig. 6b). While LMW-IR700 exhibited light-dependent cytotoxicity against LNCaP cells comparable to that of antibody-IR700 conjugates, a different morphology of bleb formation was observed after light irradiation. This morphological difference suggests that LMW-IR700 may mediate cell death through a biophysical mechanism different from the macromolecular aggregation-driven rupture observed in conventional antibody-based NIR-PIT.^120^

Cyclic peptides have also been used as delivery vehicles for NIR-PIT. The conformational constraint of cyclic peptides minimizes the entropic loss upon binding, and this results in high target affinity. Peptide 1 was designed to bind to the dimerization arm of EGFR.^121,122^ Initially, a dimeric version of Peptide 1 labeled with IR700, compound 10, was developed (Fig. 6c). However, this compound failed to induce the characteristic blebbing associated with NIR-PIT in EGFR-expressing A431 cells, probably due to the steric hindrance caused by the bulkiness of IR700. To overcome this, compound 13 was developed with a longer linker (Fig. 6d). This compound exhibited an 8-fold increase in binding rate and greater light-dependent cytotoxicity compared to compound 10. The observation of bleb formation with compound 13 demonstrates the feasibility of utilizing cyclic peptides for NIR-PIT.^123^

Otani *et al.* developed a WL12-IR700 conjugate by labeling the PD-L1-targeting peptide WL12 with IR700 for NIR-PIT (Fig. 6e).^124^ They demonstrated light-dependent killing of PD-L1-expressing cancer cells. Furthermore, *in vivo* studies showed that WL12-IR700 with irradiation achieved significant tumor regression and prolonged overall survival compared to the control and drug-only groups. Consequently, they proposed that the WL12-IR700 conjugate is a promising candidate for peptide-based NIR-PIT.

In addition, bcMT1-IR700 was synthesized by conjugating IR700 to the high-affinity bicyclic peptide bcMT1, which targets membrane-type 1 matrix metalloproteinase (MT1-MMP), a membrane-tethered protease involved in tumor invasion and metastasis (Fig. 6f). This study represents the first demonstration of NIR-PIT using a bicyclic peptide, and its antitumor effects were demonstrated both *in vitro* and *in vivo*.^125^

### Cytokines and protein carriers

Cytokines also show potential as carriers for NIR-PIT dyes. Cytokines are low-molecular-weight soluble proteins (6 to 70 kDa) secreted by various cells. They are unable to cross the cell membrane, but bind specifically to cell-surface receptors, making them useful targeting vehicles. In studies using mouse colorectal cancer MC38 cells transfected with the human IL-15 receptor alpha (MC38-hIL15Rα), an IL15-IR700 conjugate induced selective cytotoxicity upon photoirradiation. In mouse models, intratumoral injections of IL15-IR700 provided higher targeting efficiency than intravenous administration, leading to tumor growth inhibition and the induction of immunogenic cell death. These findings suggest that IR700-conjugated cytokines could be an effective therapeutic modality.^126^

Galactose-modified serum albumin (GSA) binds to β-d-galactose receptors, which are overexpressed on the cell surfaces of many cancers, including ovarian cancer, and is rapidly internalized after binding. Using GSA-IR700, it was demonstrated that β-d-galactose receptors can be targeted in a disseminated peritoneal carcinoma model with SHIN3 cells.^127^

## Development of NIR fluorescent dyes for NIR-PIT

### Design requirements and strategies

Although NIR-PIT is expected to be applicable to a wide range of diseases due to its high therapeutic efficacy and versatility, it still faces several significant challenges. For example, the penetration depth of NIR light in tissues is limited to approximately a few centimeters, making it difficult to apply this therapy to deep-seated tumors or large tumor masses.^128–130^ Further, high-intensity light irradiation is associated with edema in clinical settings. Therefore, it is important to achieve therapeutic efficacy with the lowest feasible light dose and to use longer wavelength light than 690 nm, which is employed to activate IR700.

Thus, there is a need to develop new NIR dyes for NIR-PIT with improved photochemical properties, longer-wavelength absorption for deeper tissue penetration, and simplified synthesis. Because the synthesis of IR700 is relatively complex,^8^ the development of structurally related analogues that are easier to synthesize would also be desirable. IR700 is synthesized through a 3+1-type asymmetric phthalocyanine-forming reaction using three molecules of 1,3-diiminoisoindoline and one molecule of a substituted 1,3-diiminoisoindoline bearing a *p*-methoxybenzyl-protected alkoxy group. This reaction generally produces multiple byproducts and requires difficult purification procedures. In addition, the subsequent synthesis of the hydrophilic axial ligands and the linker is relatively lengthy. After introduction of butoxy axial ligands to the phthalocyanine core, the *p*-methoxybenzyl protecting group of the linker moiety is removed, followed by substitution with siloxy axial ligands, which are further extended to introduce hydrophilic sulfonate groups. The linker is also extended and functionalized with a succinimidyl ester for antibody conjugation, ultimately yielding IR700.^8^ To date, only a limited number of phthalocyanine derivatives have been specifically engineered for NIR-PIT (Table 3), highlighting the need for broader structure–activity relationship studies and more diverse molecular scaffolds in this field.

**Table 3:** Photophysical properties of silicon phthalocyanine derivatives

Compound	*λ*_abs_ a (nm)	*λ*_em_ b (nm)	*ε* c (L mol^−1^ cm^−1^)	*Φ*_fl_ d	*Φ*_Δ_ e	Ref.
IR700	690^f^	703^f^	170 000^f^	0.28^f^	0.26^f^	132 and 134
Compound 1	677^f^	684^f^	190 000^g^	0.31^f^	—	11 and 131
Compound 2	684^f^	690^f^	170 000^g^	0.30^f^	—	131
Compound 3	691^f^	699^f^	190 000^g^	0.27^f^	—	131
Compound 4	689^f^	695^f^	170 000^g^	0.29^f^	—	131
NHS-NuSiPc	673^h^	675^h^	146 000^h^	0.27^i^	0.40^j^	132
NHS-CyaSiPc	673^h^	673^h^	193 000^h^	0.34^i^	0.45^j^	132
NHS-PySiPc	673^h^	676^h^	123 000^h^	0.14^i^	0.39^j^	132
SiPc-1	681^i^	694^i^	224 000^i^	0.29^i^	0.11^i^	133
KA800 COOH	774^f^	792^f^	140 000^f^	0.13^f^	—	134
KA800x	789^f^	818^f^	170 000^f^	0.06^f^	0.12^f^	134
WB692-CB2	692^g^	703^g^	116 000^g^	0.072^g^	0.21^k^	135

a Absorption maximum.

b Fluorescence emission maximum.

c Molar extinction coefficient.

d Fluorescence quantum yield.

e Singlet oxygen production quantum yield.

f Measured in 0.1 M sodium phosphate buffer (pH 7.0).

g Measured in H_2_O.

h Measured in DMSO.

i Measured in Dulbecco's phosphate-buffered saline (pH 7.4).

j Measured in D_2_O.

k Measured in 50 mM phosphate buffer (pH 7.5).

### Tuning IR700 analogs by varying axial ligands and linker chemistry

**Fig. 7:**
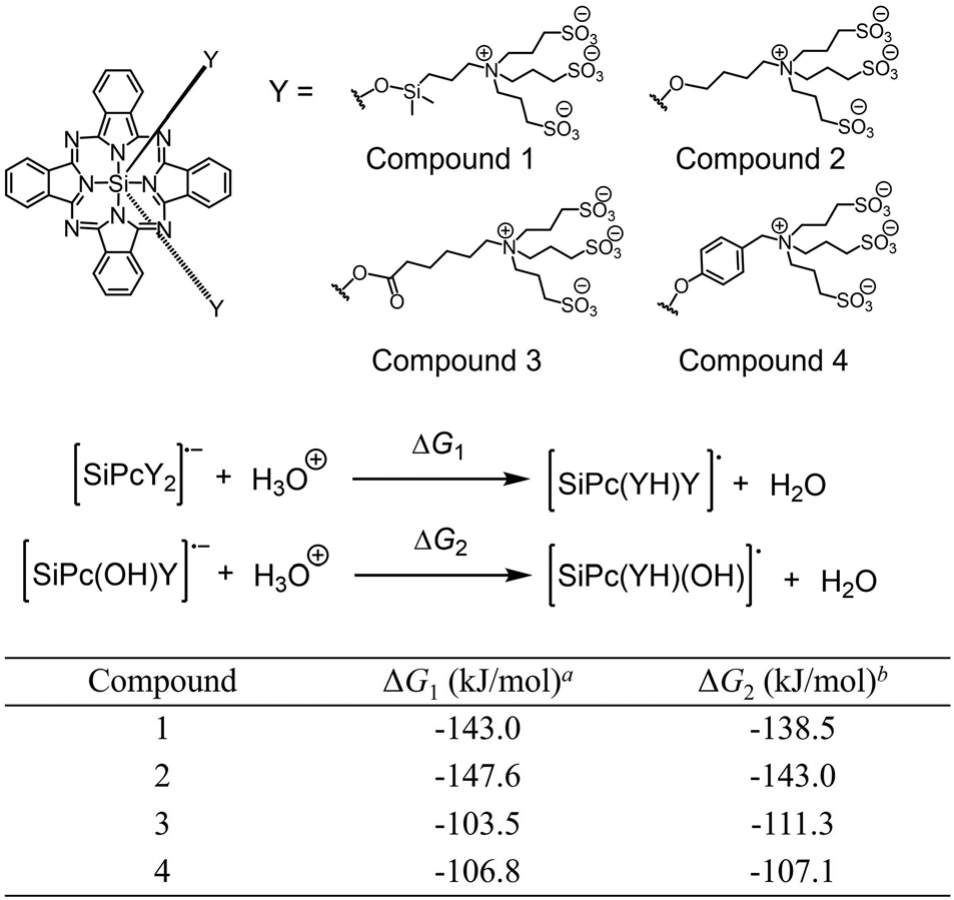
Hydrophilic silicon phthalocyanine compounds with axial ligands of various basicity. ^*a*,*b*^ Calculated Δ*G* (at 298.15 K, 1 atm) of protonation at the oxygen atom on the Si–O bond of the first axial ligand (a) and the other axial ligand (b) (ULC-BLYP/cc-pVDZ, diffuse functions were applied to N and O atoms, in water as a solvent).

To optimize the structure of IR700, research has focused on its axial ligands. As described above, the axial-ligand cleavage in IR700 requires transition to a triplet excited state, acceptance of one electron from an electron donor, and then protonation (Fig. 1b). Takakura *et al.* focused on the protonation step and developed compounds 1–4, which possess axial ligands with different basicity (Fig. 7). Measurement of their photophysical properties revealed that these compounds exhibit very similar characteristics, indicating that variation of the axial ligands has a negligible effect on the efficiency of ligand cleavage.^131^ Moreover, the Gibbs free energy change for the protonation of each radical anion was calculated using density functional theory (DFT). The results suggested that the susceptibility to protonation follows the order of alkoxy (compound 2), siloxy (compound 1), phenoxy (compound 4) and carboxy (compound 3) groups. Compound 1 is a simplified structure of IR700 and served as the control. When the axial-ligand cleavage was evaluated in the presence of dithiothreitol (DTT) as a reducing agent, the cleavage efficiency correlated well with the DFT calculation results (Fig. 7);^131^ the higher the basicity of the axial ligand, the higher the cleavage efficiency.

In contrast, the Gibbs free energy change (Δ*G*) for the protonation of each radical anion after cleavage of the first axial ligand suggested that the ease of protonation follows the order of alkoxy, siloxy, carboxy and phenoxy groups (Fig. 7). The Gibbs free energy change for the second cleavage reaction was higher than that of the first reaction, suggesting that the second protonation step is more energetically demanding and thus less likely to occur.^131^

**Fig. 8:**
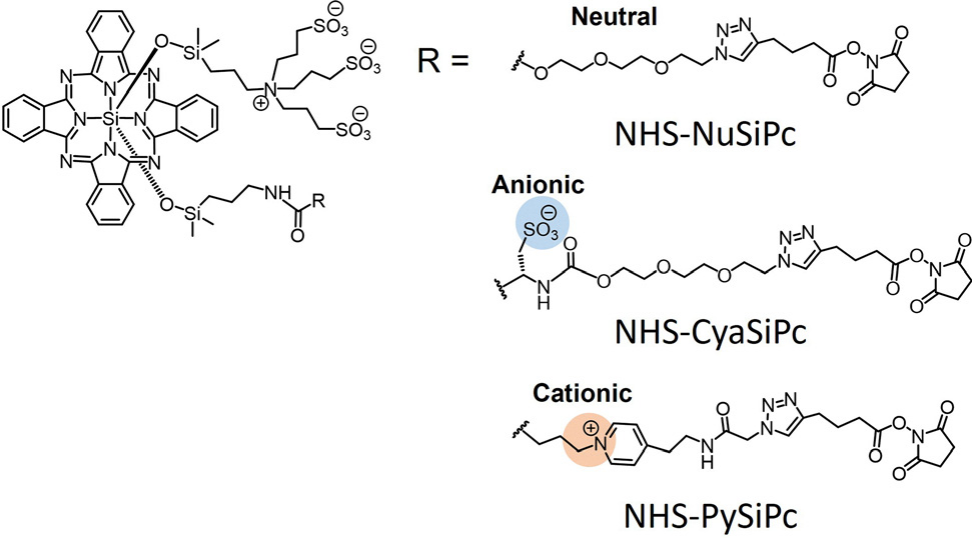
Hydrophilic silicon phthalocyanine compounds with an axial ligand substituted with a linker.

Takahashi *et al.* developed several IR700 analogues by utilizing one of the axial ligands of silicon phthalocyanine as a linker to simplify the chemical structure compared to the original IR700.^132^ This modification reduced the number of synthetic steps. While IR700 requires 11 steps, these compounds can be synthesized in just 5 steps. They synthesized three compounds: NHS-NuSiPc (neutral linker), NHS-CyaSiPc (negatively charged linker) and NHS-PySiPc (positively charged linker) (Fig. 8). Measurement of their photophysical properties showed that these molecules exhibited a slight blue shift in their fluorescence spectra (Table 3). They then labeled trastuzumab with these compounds to produce Ab-NuSiPc, Ab-CyaSiPc and Ab-PySiPc. While Ab-PySiPc showed aggregation after the labeling process, Ab-NuSiPc and Ab-CyaSiPc remained stable in solution. Ab-NuSiPc and Ab-CyaSiPc both induced cell death concentration-dependently, but their efficacy was inferior to that of Ab-IR700.^132^

### β-Substituted SiPc analogues and red-shifted derivatives

**Fig. 9:**
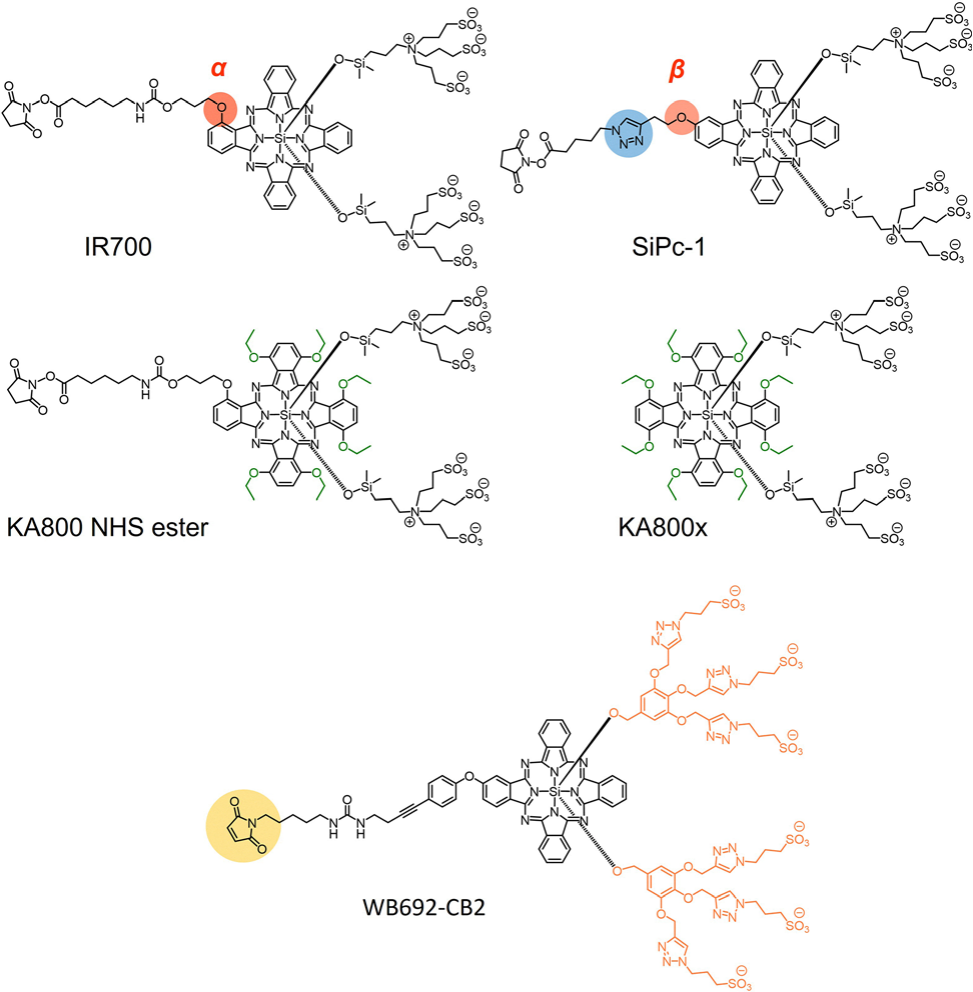
Silicon phthalocyanine derivatives based on IR700 for NIR-PIT.

Our research group has developed SiPc-1 as a novel IR700 analog. By relocating the linker from the alpha-position to the beta-position of the phthalocyanine ring, SiPc-1 effectively reduces the steric repulsion encountered during the synthesis of the conventional IR700 core (Fig. 9). Further, the use of copper-catalyzed azide–alkyne cycloaddition to facilitate linker extension provides flexibility to optimize the linker length. This structural design enables a more efficient synthetic route, reducing the number of synthetic steps from 11 for IR700 (starting from phthalonitrile) to 8.

To evaluate the therapeutic ability of SiPc-1, we labeled the anti-EGFR antibody cetuximab with SiPc-1 (cet-SiPc-1). Upon irradiation with 690 nm NIR light, cet-SiPc-1 induced a light-dose-dependent cytotoxicity and bleb formation of A431 epithelial cancer cells and DiFi colorectal cancer cells. These results demonstrated that SiPc-1 triggers cell death through a biophysical mechanism identical to that of IR700. We also labeled the anti-CD25 antibody basiliximab with SiPc-1. Upon NIR light exposure, the basiliximab-**SiPc-1** conjugate enabled selective depletion of HTLV-1-infected cells in blood samples from patients with adult T-cell leukemia (ATL). These findings support the practical utility of silicon phthalocyanines with beta-position substitution and copper-catalyzed azide–alkyne cycloaddition-based linker construction for NIR-PIT.^133^

Goto *et al.* developed a red-shifted IR700 derivative, KA800-NHS ester. The Q-band absorption of phthalocyanines in the NIR region is attributed to the transition from the HOMO to either the LUMO or LUMO+1 energy level. So, the absorption wavelength corresponds to the HOMO–LUMO energy gap. Theoretically, a red shift can be achieved by either destabilizing the HOMO energy level or stabilizing the LUMO energy level. KA800-NHS ester is a molecule in which ethoxy groups are substituted at six alpha-positions of the IR700 core (Fig. 9). Although this substitution destabilized the HOMO energy level and successfully shifted the maximum absorption wavelength from 690 nm (**IR700**) to 774 nm, KA800-NHS ester failed to induce cytotoxicity under NIR-PIT conditions. However, KA800-NHS ester conjugated with the anti-HER2 antibody trastuzumab (Ab-KA800) was cytotoxic to HER2-gene-transfected NIH3T3 (3T3-HER2) cells. Microscopic observation revealed that Ab-KA800 did not induce the cell swelling or bleb formation typically associated with NIR-PIT, but nevertheless, light-dependent cytotoxicity was still observed. Thus, Ab-KA800-induced cell death is thought to be primarily mediated by singlet oxygen generation rather than membrane rupture.^134^ In the case of KA800-NHS ester, the strategy of destabilizing the HOMO energy level led to a decrease in the efficiency of radical anion formation, thereby preventing the initiation of the NIR-PIT reaction. This indicates that, for the development of effective photosensitizers, stabilizing the LUMO energy level is more desirable than destabilizing the HOMO energy level.^134^

Wolf *et al.* developed WB692-CB2, which contains a maleimide group for conjugation and enables selective conjugation to cysteine residues (Fig. 9). The NHS ester of IR700 results in non-selective covalent modification of lysine residues on antibodies, whereas WB692-CB2 allows site-specific labeling at cysteine residues, facilitating the preparation of more homogeneous antibody–drug conjugates. This molecule also differs from IR700 in that the linker substitution position is changed from alpha to beta, sulfo groups are introduced *via* copper-catalyzed azide–alkyne cycloaddition and the linker is extended through Sonogashira coupling. When WB692-CB2 was conjugated to the humanized anti-PSMA antibody h3/F11.19, the conjugate induced NIR-PIT effects in prostate cancer cell lines expressing PSMA (LNCaP and C4-2 cells) upon NIR light irradiation at 24 hours after the antibody treatment.^135^

In terms of molecular design, researchers are seeking to establish simplified synthetic routes and to achieve a bathochromic shift (longer absorption wavelengths) to enable access to deep-seated tissues. However, no analog yet shows greater cytotoxicity than IR700. Furthermore, despite the advances in NIR-PIT, the reduction of treatment-induced inflammation remains a challenge for clinical implementation. Thus, to promote the clinical adoption of this therapy, we will require NIR dyes with greater therapeutic activity than IR700, as well as optimization of photophysical properties, and the establishment of efficient dye-labelling techniques. Continued progress in these areas can be expected to expand the potential applications of NIR-PIT.

## Conclusions

This review deals with the fundamental principles and clinical applications of NIR-PIT using IR700, together with recent developments in the design of novel photosensitizers for NIR-PIT. The core of this technology lies in the unique photochemical behavior of silicon phthalocyanines: light irradiation triggers cleavage of the axial ligands, causing a marked change in the molecule's physicochemical properties from hydrophilic to highly hydrophobic. This transition induces physical damage to the cell membrane, leading to rapid cell death. This process also induces immunogenic cell death, which facilitates activation of the host's anti-tumor immune response, and also shows synergy with immune checkpoint inhibitors, suggesting considerable potential for future clinical oncology. Moreover, the utility of NIR-PIT extends beyond oncology, since recent studies targeting viruses and bacteria have indicated its potential to treat other diseases.

In terms of molecular design, researchers are seeking to establish simplified synthetic routes and to achieve a bathochromic shift (longer absorption wavelengths) to enable access to deep-seated tissues. However, no analog yet shows greater cytotoxicity than IR700. Furthermore, despite the advances in NIR-PIT, the reduction of treatment-induced inflammation remains a challenge for clinical implementation. Thus, to promote the clinical adoption of this therapy, we will require NIR dyes with greater therapeutic activity than IR700, as well as optimization of photophysical properties, and the establishment of efficient dye-labelling techniques. Continued progress in these areas can be expected to expand the potential applications of NIR-PIT.

## Conflicts of interest

There are no conflicts to declare.

## Data Availability

No primary research results, software or code have been included and no new data were generated or analysed as part of this review.
